# Genetic Variation in the Dopamine System Influences Intervention Outcome in Children with Cerebral Palsy

**DOI:** 10.1016/j.ebiom.2017.12.028

**Published:** 2018-01-09

**Authors:** Rochellys Diaz Heijtz, Rita Almeida, Ann Christin Eliasson, Hans Forssberg

**Affiliations:** a Department of Neuroscience, Karolinska Institutet, Stockholm, Sweden; b Department of Women's and Children's Health, Karolinska Institutet, Astrid Lindgren Children's Hospital, Stockholm, Sweden

**Keywords:** Cerebral palsy, Dopamine genes, Intervention, Hand motor assessment

## Abstract

**Background:**

There is large variation in treatment responses in children with cerebral palsy. Experimental and clinical results suggest that dopamine neurotransmission and brain-derived neurotrophic factor (BDNF) signalling are involved in motor learning and plasticity, which are key factors in modern habilitation success. We examined whether naturally occurring variations in dopamine and BDNF genes influenced the treatment outcomes.

**Methods:**

Thirty-three children (18–60 months of age) with spastic unilateral cerebral palsy were enrolled in the study. Each child had participated in a training programme consisting of active training of the involved hand for 2 h every day during a 2-month training period. The training outcome was measured using Assisting Hand Assessment before and after the training period. Saliva was collected for genotyping of COMT, DAT, DRD1, DRD2, DRD3, and BDNF. Regression analyses were used to examine associations between genetic variation and training outcome.

**Findings:**

There was a statistically significant association between variation in dopamine genes and treatment outcome. Children with a high polygenic dopamine gene score including polymorphisms of five dopamine genes (COMT, DAT, DRD1, DRD2, and DRD3), and reflecting higher endogenous dopaminergic neurotransmission, had the greatest functional outcome gains after intervention.

**Interpretation:**

Naturally occurring genetic variation in the dopamine system can influence treatment outcomes in children with cerebral palsy. A polygenic dopamine score might be valid for treatment outcome prediction and for designing individually tailored interventions for children with cerebral palsy.

## Highlights

• Naturally occurring variation of dopamine genes is associated with treatment outcomes in children with cerebral palsy.
• Children with polymorphisms reflecting higher endogenous dopaminergic neurotransmission had the greatest functional gains.
• A polygenic dopamine score might be valid to predict treatment outcome.

New evidence-based therapies including active motor learning and training for children with cerebral palsy improve motor function at a group level, but there are also large inter-individual variations. Naturally occurring variations in dopamine and BDNF genes affect motor learning and cortical plasticity.

This study showed that naturally occurring genetic variation of five dopamine genes was associated with the outcome of a 2-month long active upper limb motor training intervention in children with unilateral cerebral palsy. The results suggest that a polygenic dopamine gene score can be used to predict the outcome of motor training programmes for children with cerebral palsy.

## 1 Introduction

A plethora of experience-derived treatments for cerebral palsy have been developed over time. Researchers and clinicians have only during the past few decades adopted an evidence-based approach to identify and use effective interventions (Novak et al., 2013). New therapies based on active motor learning and motor training have been found to improve motor function and activity (e.g., modified constraint-induced movement therapy, bimanual training, and goal-directed training) (Eliasson et al., 2005, Eliasson et al., 2011, Gordon et al., 2007, Ketelaar et al., 2001). A common problem with these studies is that they focus on main effects at the group level, but neglect the effects of individual differences (Damiano, 2014). Due to large inter-individual variation in treatment response, there are often barely significant differences between the study groups (see Figs. 2 and 4 in (Chiu and Ada, 2016) and Fig. 3 in (Eliasson et al., 2005)). An intervention that is effective for one child with cerebral palsy may not be effective for another child.

These observed large inter-individual differences in treatment outcomes have negative consequences for non-responders because time and effort will be expended without any gains in functional improvement. Identification of the causes of the variability is an important step in the advancement of personalized rehabilitation medicine. Each child with cerebral palsy can then receive an individually tailored intervention. The concept of personalized medicine has evolved mainly from the variability observed in response to various drugs. It has developed into the field of pharmacogenetics, which examines how genetic differences (especially in metabolic pathways) affect individual responses to drugs (Roses, 2000). The genetic effects may not be as influential for therapeutic interventions in children with cerebral palsy. However, identification of factors that predict the individual's response to an intervention would be useful for the patient and for the health providers attempting to optimize care.

Several factors may influence the outcome of interventions in cerebral palsy, which encompasses heterogeneous clinical phenotypes and aetiologies. Factors that can affect the outcome of an intervention can be associated with the white and/or grey matter brain injuries of varying location, size, and time of origin. These conditions are known to affect cognitive and motor functions. Many children have no detectable risk factors, and 30% of the cases of cerebral palsy may be of genetic origin (Fahey et al., 2017). However, the contribution of genetic variation to treatment outcomes remains mostly unknown.

Functional genetic variation can influence motor learning and the cortical plasticity that form the principle foundation for modern rehabilitation interventions. One of the best-characterized examples is the functional val^66^met polymorphism in the gene for brain derived neurotrophic factor (BDNF). BDNF is highly expressed throughout the brain and has important roles in development, plasticity, and repair. The presence of the BDNF val^66^met polymorphism is associated with poor short-term motor learning gains and altered short-term cortical plasticity (McHughen et al., 2010). Results of animal studies indicate that microglial BDNF has an important physiological function in motor learning via promotion of learning-related synapse formation (Parkhurst et al., 2013).

Dopamine signalling is an essential component of various brain functions (e.g., motor control, reward, learning, and plasticity) (Chudasama and Robbins, 2006). Rodent studies have found that mesocortical dopaminergic pathways from the ventral tegmental area to the motor cortex are involved in skilled motor learning and associated synaptic plasticity (Molina-Luna et al., 2009). This result suggests that the motor cortex requires an optimal level of dopamine for learning new motor skills The role of dopamine signalling is further corroborated by the association between acquisition of new motor skills and changes in the intracellular cAMP/PKA/DARPP-32 pathway; inhibition of this pathway impairs motor learning (Qian et al., 2015). A naturally occurring genetic variation in the rodent mesocortical dopamine system parallels differences in motor skill learning and plasticity (Qian et al., 2013). Results of human studies indicate that functional polymorphisms in genes encoding for dopamine receptors, and dopamine transporter and degradation enzymes, contribute to inter-individual differences in learning and cognitive performance. Polymorphisms that reduce dopamine transmission are associated with poorer function (Pearson-Fuhrhop et al., 2013, Baetu et al., 2015, Noohi et al., 2016, Noohi et al., 2014, Huertas et al., 2012). Pearson-Fuhrhop et al. (2013) found that genetic variation in the human dopamine system affects motor learning outcomes in healthy adults. A gene score reflecting the collective effects of five dopamine polymorphisms associated with synaptic dopamine availability (COMT and DAT) and dopamine receptor binding (DRD1, DRD2, DRD3) was an important contribution of this study. The authors found that individuals with the higher dopamine scores that corresponded to higher dopaminergic neurotransmission also had significantly greater motor learning rates.

The results of human and animal studies thus suggest that dopamine and BDNF are involved in motor learning, and that variation in dopamine and BDNF genes might contribute to the inter-individual differences in treatment response. The aim of this study was to examine the influences of functional genetic variation in the dopamine system and BDNF on the outcome of an intervention programme for children with cerebral palsy. The intervention used was modified Constraint Movement Therapy (CIMT) (Eliasson et al., 2005, Eliasson et al., 2011), which is based on active motor learning and motor training.

## 2 Methods

### 2.1 Participants

The participants were recruited from two previous intervention studies of children with spastic unilateral cerebral palsy. The first study was a controlled clinical trial that included 21 children (18–48 months of age) (Eliasson et al., 2005). The second study used a randomized crossover design that included 25 children (18–60 months of age) (Eliasson et al., 2011). Each child underwent an Assisting Hand Assessment (AHA) before and after the intervention and had complied with the scheduled training program. Each of the 46 children was invited to participate in this study 6–15 years after the first two trials, when they were asked to provide a saliva sample for genetic analysis. Thirty-five subjects accepted the invitation. The age, sex, and AHA-unit at baseline or after training characteristics did not differ between those who accepted versus those who did not respond to, or refused, the invitation to participate (n = 11).

Each participant provided written informed consent prior to the collection of the saliva sample. The study was approved by the Regional Ethical Review Board in Stockholm (Dnr: 2015/61–31/2).

### 2.2 Intervention

The training programme for both studies comprised active training of the involved hand for 2 h each day during a 2-month training period. Toys and activities relevant for the age and ability of the child were used. During this modified Constraint-Induced Movement Therapy (CIMT), a comfortable fabric glove with a built in volar stiff plastic splint was worn on the less impaired hand to encourage each child to use the impaired hand (Eliasson et al., 2005, Eliasson et al., 2011). Parents and school teachers acted as treatment providers after attending an introductory educational and training session. One therapy session per week was supervised by a therapist. The results of each training session were recorded in a log book.

### 2.3 Assessment

The change in the Assisting Hand Assessment (AHA) (Krumlinde-Sundholm et al., 2007, Holmefur et al., 2007, Holmefur and Krumlinde-Sundholm, 2016, Holmefur et al., 2009) performed before and after the intervention period was used as the primary outcome of the CIMT intervention. Each assessment consisted of a 15-min long, semi-structured, video-recorded play session with toys requiring bimanual manipulation. The bimanual activity was scored for 22 items using a 4-point rating scale. The raw scores were converted to logits using Rasch analysis and transformed to a 0- to 100-unit scale (Holmefur et al., 2009). Each video recording was scored by a blinded evaluator who did not know the children, group allocation, or time of assessment (before versus after intervention).

In this study, we used the change in AHA-units (i.e., after CIMT minus before CIMT) as the primary outcome variable to examine the effect of genetic variation on intervention outcome.

### 2.4 Genetic Analysis

Saliva samples were collected from 35 children with spastic unilateral cerebral palsy using the Oragene DNA Sample Collection Kit (Genotek, Ottawa, Ontario, Canada). DNA was extracted within 3 days of collection following the manufacturer's protocol. DNA quantity and quality were evaluated using a NanoDrop TM 2000 spectrophotometer (NanoDrop Technologies, Wilmington, DE, USA). Data from 33 participants were included in the analysis because two poor-quality saliva DNA samples were excluded. All samples were stored at − 20 °C until use. Genotype was determined for COMT rs4680, DRD1 rs4532, DRD2/ANKK1 rs1800497, DRD3 rs6280, DAT1 VNTR, and BDNF rs6265 using polymerase chain reaction (PCR)-restriction fragment length polymorphism analysis as previously described (Pearson-Fuhrhop et al., 2013). All saliva DNA samples were genotyped by an investigator blinded to the subjects' identity.

A combined “polygenic” dopamine gene score was determined as previously described (Pearson-Fuhrhop et al., 2013). Briefly, this score represented the cumulative effects of five dopamine-related polymorphisms with established biological functional effects on dopamine neurotransmission. Each genotype associated with low dopamine signalling received a score of zero; each genotype with a high signal received a score of one. The sum of the five individual genotypes ranged from zero (lowest dopamine neurotransmission) to five (highest dopamine neurotransmission).

### 2.5 Statistics

All statistical analyses were performed using R (version 3.2.3). In the main analysis, we used a general linear model to explain the changes in AHA-units in terms of the following variables used together: combined dopamine gene score (0–5, considered as a continuous variable), BDNF gene score (0 for VAL/MET and MET/MET or one for VAL/VAL), and sex and age (months at the start of the 2-month intervention period). In subsequent explorations of single-dopamine gene relationships, a four-gene score (i.e., successively leaving out one dopamine gene at a time) was inserted into the same model used with the multi-gene score, and each single gene score, to evaluate each dopamine gene independently of the other genes. Finally, in a separate linear model, we evaluated the effect of AHA-units at baseline on the changes in AHA-units, while maintaining all other explanatory variables. Chi-Square and exact tests were used to assess the Hardy-Weinberg equilibrium. The significance level for effects was set at p < 0·05.

## 3 Results

The results for the characteristics of the 33 children with spastic unilateral cerebral palsy are presented in Table 1. There was considerable variation in baseline AHA-units and in changes in AHA-units during the intervention period.

**Table 1:** Characteristics of participants with spastic unilateral cerebral palsy.

Age (months) at intervention	26 (18–55)
Participants age; ≤ 24 months/>24 months	13/20
Gender; Male/Female	21/12
Impaired side; Right/Left	21/12
Gestational weeks at birth	39 (27–42)
AHA-units at baseline	50 (7–81)
AHA-units change	6 (− 3 − + 18)

Values are presented as numbers of subjects and as median and total range of the sample. AHA = Assistive Hand Assessment units (0–100 unit scale) before, and change between before and after intervention.

The results for the distributions of alleles for the six genes in the 33 children with cerebral palsy are presented in Table 2. The allelic distributions of all six genes were in Hardy-Weinberg equilibrium (p > 0.193).

**Table 2:** Genotypes, alleles, and allele-carrier frequencies of BDNF and dopamine genes.

Gene	Genotype	Type	N	%
*COMT* (rs4680)	Val158Met	Val/Val	6	18·2%
		Val/Met	20	60·6%
		Met/Met	7	21·2%
	Allele carrier	Met (+)	27	81·8%
		Met (−)	6	18·2%
*DRD1* (rs4532)	A-48G SNP	AA	5	15·2%
		AG	14	42·4%
		GG	14	42·4%
	Allele carrier	G (+)	28	84·8%
		G (−)	5	15·2%
*DRD2* (rs1800497)	Glu713Lys	Glu/Glu	21	63·6%
		Glu/Lys	11	33·3%
		Lys/Lys	1	3·0%
	Allele carrier	Lys (+)	12	36·4%
		Lys (−)	21	63·6%
*DRD3* (rs6280)	Ser9Gly	Ser/Ser	16	48·5%
		Ser/Gly	12	36·4%
		Gly/Gly	5	15·2%
	Allele carrier	Gly (+)	17	51·5%
		Gly (−)	16	48·5%
*DAT* (rs28363170)	40 bp VNTR	4/4	1	3·0%
		9/9	2	6·1%
		9/10	9	27·3%
		10/10	19	57·6%
		10/11	1	3·0%
		11/11	1	3·0%
	Allele carrier	9-repeat (+)	11	33·3%
		9-repeat (−)	22	66·6%
*BDNF* (rs6265)	Val66Met	Val/Val	22	66·7%
		Val/Met	9	27·3%
		Met/Met	2	6·1%
	Allele carrier	Met (+)	11	33·3%
		Met (−)	22	66·7%

COMT, Catechol-*O*-methyl transferase; DRD1, dopamine receptor D1; DRD2, dopamine receptor D2; DRD3, dopamine receptor D3; DAT, dopamine transporter; BDNF; brain-derived neurotrophic factor.

The results for the distribution of dopamine score in relation to sex, age, and AHA-units at baseline are presented in Table 3. There was no correlation between dopamine gene score, and age or AHA units.

**Table 3:** Sex, age, and AHA-unit distribution per polygenic dopamine gene score unit.

	All	DA score 0	DA score 1	DA score 2	DA score 3	DA score 4	DA score 5	p
N	33	0	3	9	4	14	3	
Male	21		3	4	4	9	1	
Female	12		0	5	0	5	2	
Age			24·3 ± 2·9	26·7 ± 5·4	28·5 ± 11·6	33·1 ± 11·6	23·7 ± 7·4	0·262
AHA			38·7 ± 23·1	59·0 ± 10·7	34·5 ± 33·0	43·0 ± 13·9	36·0 ± 21·9	0·184

Mean and SD values are given. p-values are for the correlation between age or baseline AHA unit and dopamine (DA) score.

**Fig. 1:**
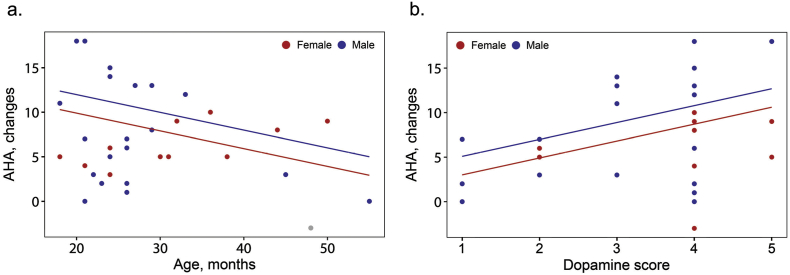
Association between intervention improvement as measured by changes in AHA units and age (a) and dopamine gene score (b). In both panels the dots represent individual measurements of males and females, respectively. When estimating a regression line, all independent variables not represented in each plot were fixed at their median values or at their most common value in case of categorical variables.

The effect of genetic variation, sex, and age on training outcome was examined using a general linear model. We used the change in AHA-units as the outcome variable; age, sex, dopamine gene score, and BDNF were considered together as independent variables. Age and dopamine score had statistically significant effects on training outcome (parameter estimate − 0.199, p = 0.042 for age; parameter estimate 1.897, p = 0.010 for dopamine score). The results for BDNF and sex were not statistically significant (p = 0.229 for BDNF; p = 0.245 for sex). In Fig. 1 the relationship between changes in AHA and the variables dopamine score and age is illustrated for both males and females.

Next, we explored the individual contributions of the five dopamine genes involved in the combined dopamine gene score by removing one gene at a time or by calculating the effect of only one gene at a time. The results are presented in Table 4. The Akaike Information Criteria (AIC) result indicated that the model with the original five-dopamine gene score was a better fit to the data compared with the other models (AIC = 200.621), except for the model excluding the DRD3 gene (AIC = 198.989). The effect of the five-dopamine score was also more significant in the original model (p = 0.010) than in the other models, except for the four-dopamine gene score excluding DRD3 (p = 0.005). Elimination of DRD1 and DAT resulted in less significant dopamine scores (p = 0.030 and p = 0.026, respectively). The effects of DRD1 (p = 0.070) and DAT (p = 0.056) alone were not statistically significant.

**Table 4:** Linear model analysis using different dopamine scores.

Dopamine score	AIC	Parameter estimate	p value
5-gene score	200.621	1·897	0.010
4-gene score without DRD1	202.871	1·785	0.030
4-gene score without DRD2	201.554	2·269	0.016
4-gene score without DRD3	198.989	2·306	0.005
4-gene score without COMT	202.270	1·906	0.023
4-gene score without DAT	202.568	2·121	0.026
DRD1 only	204.568	4·650	0.070
DRD2 only	205.621	2·946	0.122
DRD3 only	208.455	0·343	0.858
COMT only	205.722	3·710	0.128
DAT only	204.096	3·630	0.056

The dependent variable was the change in AHA-units from baseline to after the intervention. Various dopamine gene scores were used as explanatory variables together with age, sex, and BDNF gene score. Dopamine four-gene scores were formed by removing each single-gene contribution, and the contribution of each gene was calculated by using only that single gene in the model. The table shows the Akaike Information Criteria (AIC) for each model (lower values correspond to a better model), coefficient estimates for the dopamine score variables, and corresponding p-values.

We then explored whether the AHA-unit value at baseline had an effect on outcome. We used the linear model described above and added the values for the AHA-units before training as an explanatory variable. The effect of the dopamine five-gene score remained statistically significant (parameter estimate 1.435, p = 0.046) even after controlling for AHA-units at baseline. The effect of AHA-units at baseline was also statistically significant (parameter estimate − 0.096, p = 0.043). However, the result using this model indicated that the effect of age was no longer statistically significant, but there was a tendency towards significance (parameter estimate − 0.158, p = 0.093). The effects of sex and BDNF remained statistically insignificant (p = 0.162 and p = 0.471, respectively).

## 4 Discussion

The results of this study are the first to suggest that naturally occurring genetic variation can predict the outcome of a rehabilitation intervention for patients with movement disorders. The results indicated that there was a statistically significant association between a polygenic dopamine gene score reflecting the collective effects of five dopamine gene polymorphisms (COMT, DAT, DRD1, DRD2, and DRD3), and CIMT outcome in 33 children with spastic unilateral cerebral palsy. Consistent with our hypothesis, children with a high gene score, and thus higher endogenous dopaminergic neurotransmission, had the greatest functional outcome gains after intervention. The gains were measured using changes in AHA units. We found that there was no statistically significant effect of the BDNF val66met polymorphism on intervention outcome. The results thus suggested that a polygenic dopamine gene score can be useful for predicting the outcome of motor interventions in individuals with motor disabilities.

The association between dopamine neurotransmission (as reflected by the polygenic dopamine gene score) and functional CIMT outcome in children with spastic unilateral cerebral palsy likely reflects a more general principle of dopamine-dependent brain plasticity. This finding may therefore be relevant for other ages and other types of movement disorders. The association suggests that the effectiveness of interventions based on active motor learning and training depends on dopamine-mediated regulation of cortico-striatal plasticity. Abundant experimental evidence indicates that dopamine has a key role in motor learning and associated cortical plasticity. Rodent studies have found that mesocortical dopaminergic transmission is required for learning new motor skills, but not for executing learned movements (Molina-Luna et al., 2009). Intracellular dopamine signalling pathways are involved in the neuroplasticity that occurs during the more active phase of motor skill learning (Qian et al., 2015) Our study using inbred mice (*C57BL*/*6 and BALB/c* mice) revealed that genetically driven variation in midbrain dopamine neurotransmission is a major contributor to individual differences in motor skill learning (Qian et al., 2013) One major challenge to understanding the effects of genetic variation on motor function in rehabilitation outcomes is that a large number of proteins affects dopamine neurotransmission and the small effect size of single nucleotide polymorphisms. Pearson-Fuhrhop et al. (2013) addressed this challenge by creating the polygenic dopamine gene score used in this study. This score combines the effect of five polymorphisms (COMT, DAT, DRD1, DRD2, and DRD3) that affect brain dopamine neurotransmission. They found that this polygenic dopamine score can be used to predict motor learning outcomes in heathy adults. Individuals with higher dopamine scores corresponding to higher dopaminergic neurotransmission levels had greater motor learning gains. We used the polygenic dopamine gene score and found that it strongly predicted intervention outcome in children with unilateral cerebral palsy (p = 0.010). Children with the higher dopamine scores that reflected higher levels of dopamine neurotransmission improved more than children with lower scores (Fig. 1). These results correspond to the clinical experience of many therapists; even among children with similar clinical phenotypes, some children with cerebral palsy respond well to an intervention but others do not. This finding is also consistent with results from intervention studies finding large inter-individual variations in the outcomes of therapies in children with cerebral palsy (Eliasson et al., 2005, Damiano, 2014, Chiu and Ada, 2016).Our results may thus have important clinical implications for understanding the variation in response to rehabilitation interventions. They suggest that genetic variation in dopamine neurotransmission may be one of the factors that affect the outcome of an intervention, and that genotyping can be useful when planning the rehabilitation.

The five proteins (COMT, DAT, DRD1, DRD2, and DRD3) involved in the polygenic dopamine gene score have different roles in dopamine neurotransmission and have different patterns of distribution in various brain regions. Therefore, these five gene polymorphisms may affect training-induced plasticity to differing degrees. We adapted the five-gene score model and examined the effects of single dopamine genes by calculating a four-gene score (i.e., successively leaving out one dopamine gene at a time). Removal of the DRD3 gene increased the strength of the association; elimination of any of the other genes (but especially DRD1 and DAT) weakened the association (Table 4). Entering DRD1 or DAT polymorphisms into the model as the single source of genetic variation tended to suggest an association with intervention outcome. These results suggested that the polymorphism of DRD3 did not contribute to the variation in intervention outcome. A combined dopamine four-gene score consisting of DRD1, DRD2, COMT, and DAT might be a better predictor of the outcome.

Our results (Table 4) also suggested that among the remaining four-dopamine polymorphisms, DRD1 and DAT are the strongest drivers of the neuroplasticity affecting outcome. Pearson-Fuhrhop et al. (2013) found that the DRD2 polymorphism had the strongest effects on motor learning and transcranial magnetic stimulation map plasticity, while COMT did not contribute to the variation. Their results are consistent with the results of a study using a procedural visuo-motor learning task in healthy young adults. The polymorphism of the DRD2 gene, but not the COMT gene, was associated with faster learning (Huertas et al., 2012). In contrast, a more recently published study found that polymorphisms in the DRD1, DRD2, and COMT genes affect different aspects of motor sequence learning (Baetu et al., 2015). Several studies on the association between dopamine gene polymorphisms and motor recovery after stroke when patients usually participate in active motor rehabilitation programs have been published. Kim et al. (2016) found that there were statistically significant associations between COMT polymorphisms and motor recovery at 3 months and 6 months after stroke; the associations with the DRD1, DRD2, and DRD3 gene polymorphisms were not statistically significant. Liepert et al. (2013) found that stroke patients with the COMT val/val polymorphism had a better outcome immediately after stroke. However, this difference in outcome was not due to more improvement, but to greater motor functions and abilities directly after the stroke. Taken together, these findings suggest that polymorphisms of different dopamine genes may affect different aspects of neuroplasticity.

Our initial analysis also found that age had a statistically significant effect on the intervention outcome (Fig. 1). Younger children had greater gains than older children. This result corresponds with the premise that neuroplasticity is stronger at younger ages, and that the dopamine-dependent plasticity of the neural circuits targeted by the active motor training is stronger at younger ages. This finding, however, contrasts with previous study findings on the efficacy of CIMT for unilateral cerebral palsy that suggest that improvements are not age-dependent (Gordon et al., 2006, Sakzewski et al., 2011). Instead, children with more impaired hand function seem to achieve greater improvements from CIMT. (Eliasson et al., 2005, Sakzewski et al., 2011) We reanalysed the data and included the baseline AHA-units (i.e., before training) as an additional independent variable in the model. The effect of AHA-units at baseline was statistically significant (p = 0.043), but the effect of age was not (p = 0.093). It seems that the age effect was driven by differences in hand motor function before the intervention; younger children performed at a lower level. Therefore, from this study it is not possible to conclude whether dopamine-dependent plasticity is age-dependent. These neuroplasticity mechanisms are probably still active in older patients with other forms of movement disorders, as recently found in studies on recovery after stroke (Kim et al., 2016, Liepert et al., 2013).

Pearson-Fuhrhop et al. (2013) also found that a polygenic dopamine score might be useful for predicting individuals whose motor learning might benefit from a dopaminergic therapy. Individuals with a lower gene score, and thus lower endogenous dopaminergic neurotransmission, had the greatest motor learning improvement when given L-DOPA, which enhances the synaptic dopamine. In contrast, the same treatment had a negative effect on individuals with a high dopamine score. This finding is consistent with previous study findings that dopaminergic modulation of frontal-striatal circuitry follows an inverted U-shaped curve. Cognition and executive functions reach peak performance at optimal levels of dopamine; too low or too high dopamine levels result in poorer performance (Williams and Goldmanrakic, 1995, Thirugnanasambandam et al., 2011) These results suggest that it is possible to augment treatment outcomes in non-responders with low endogenous dopaminergic neurotransmission by combining a motor rehabilitation program with L-DOPA or other dopamine stimulating drugs.

The strengths of this study include the use of a well-characterized cohort of children with cerebral palsy who were exposed to the same type of intervention program and assessment. The limitations of the study were the small size of the cohort and the retrospective study design. Saliva was collected for genotyping 6–15 years after the intervention. However, gene polymorphisms are stable over time and age-related changes likely did not affect the results.

In conclusion, our findings indicate the importance of naturally occurring genetic variation in the dopamine system for treatment outcomes in children with spastic unilateral cerebral palsy. We propose that the polygenic dopamine score used in the current study might be useful for prediction of treatment outcome, while it needs to be replicated in other studies before it can be translated into clinical practise. The designs of individually tailored interventions for children with cerebral palsy could benefit from application of this scoring system.

## Funding Sources

Swedish Research Council (5925), Foundation Olle Engkvist Byggmästare, Swedish Brain Foundation, Foundation Frimurarna Barnhuset, Promobilia Foundation, Strategic Research Programme of Neuroscience at Karolinska Institutet. The funding bodies had no role in planning the study, analysing the data or writing the manuscript.

## Conflict of Interest Statement

All authors declare that there are no conflicts of interest to report.

## Author Contributions

RDH and HF conceived, designed and organized the study. ACE was responsible for the intervention studies and analysed the AHA data. RDH performed the genetic analysis. RA performed the statistical analysis. HF wrote the first manuscript draft and all authors contributed to the subsequent drafts and approved the final version.
